# Long-Term Tinnitus Suppression with Linear Octave Frequency Transposition Hearing Aids

**DOI:** 10.1371/journal.pone.0051915

**Published:** 2012-12-20

**Authors:** Elisabeth Peltier, Cedric Peltier, Stephanie Tahar, Evelyne Alliot-Lugaz, Yves Cazals

**Affiliations:** 1 Laboratoire Chelles surdité, Chelles, France; 2 Laboratoire de Neurosciences Intégratives et Adaptatives, Aix-Marseille Université, CNRS UMR 7260, Féderation de Recherche 3C (Cerveau, Comportement, Cognition), Marseille, France; Emory Univ. School of Medicine, United States of America

## Abstract

Over the last three years of hearing aid dispensing, it was observed that among 74 subjects fitted with a linear octave frequency transposition (LOFT) hearing aid, 60 reported partial or complete tinnitus suppression during day and night, an effect still lasting after several months or years of daily use. We report in more details on 38 subjects from whom we obtained quantified measures of tinnitus suppression through visual analog scaling and several additional psychoacoustic and audiometric measures. The long-term suppression seems independent of subject age, and of duration and subjective localization of tinnitus. A small but significant correlation was found with audiogram losses but not with high frequency loss slope. Long-term tinnitus suppression was observed for different etiologies, but with a low success rate for sudden deafness. It should be noted that a majority of subjects (23) had a history of noise exposure. Tinnitus suppression started after a few days of LOFT hearing aid use and reached a maximum after a few weeks of daily use. For nine subjects different amounts of frequency shifting were tried and found more or less successful for long-term tinnitus suppression, no correlation was found with tinnitus pitch. When the use of the LOFT hearing aid was stopped tinnitus reappeared within a day, and after re-using the LOFT aid it disappeared again within a day. For about one third of the 38 subjects a classical amplification or a non linear frequency compression aid was also tried, and no such tinnitus suppression was observed. Besides improvements in audiometric sensitivity to high frequencies and in speech discrimination scores, LOFT can be considered as a remarkable opportunity to suppress tinnitus over a long time scale. From a pathophysiological viewpoint these observations seem to fit with a possible re-attribution of activity to previously deprived cerebral areas corresponding to high frequency coding.

## Introduction

Tinnitus, the perception of a sound without an external stimulus, often occurs together with hearing loss. It is a pathology which affects approximately 10% of the population worldwide [1]. It has long been observed that tinnitus sounds most loud and annoying in a quiet environment [2], [3] whereas noisy surroundings can produce a partial to complete suppression of tinnitus. Thus for many hearing-impaired people the use of a hearing aid besides providing improved perception of external sounds, can be beneficial against tinnitus [4], [5], [6]. Most often the tinnitus suppressing effects provided by external sounds do not last much after returning to a quiet environment, and if any, the long term suppressive effects are weak [7]. In addition, hyperacusis often associated with tinnitus [8], [9] can limit the use of amplification of external sounds which may exacerbate tinnitus perception.

Special hearing aids delivering a noise as tinnitus masker were developed several decades ago; dedicated studies report some success but overall do not bring strong evidence of benefit [10]. Masking of tinnitus is obtained for about half of the subjects [11], but the constant noise may become too bothersome [12], or can adversely interfere with the hearing of other sounds. In addition to tinnitus masking during the masker sound, many subjects report that at cessation of masking, tinnitus can remain attenuated for seconds up to a few minutes, a phenomenon called residual inhibition [13], [14].

The perceived pitch of tinnitus is very often associated with audiometric losses at the corresponding frequencies [15], [16], [17]. This association strongly suggests the hearing loss as a first source of tinnitus although it remains unknown why people with similar hearing losses are or are not affected by tinnitus. For a majority of hearing-impaired people the hearing loss affects the high frequencies and, as a correlate, tinnitus sufferers also most often report high frequency tinnitus pitches [12], [16].

When the degree of hearing loss at some frequencies is moderate or severe the quality and discriminability of sounds at these frequencies can be so deteriorated that providing amplified sounds at these frequencies do not bring benefit and may even be detrimental [18]. As information given by high frequencies can be a decisive help for sound identification and for speech intelligibility, transposition of high to lower frequencies has been tried in hearing aids for decades [19]. Up to now frequency lowering techniques continue to be a matter of studies [20], [21] and hearing aid manufacturers presently offer some algorithms. A recent study provides details upon technical differences between the two main hearing aids using frequency lowering algorithms : the Phonak SR using a non linear frequency compression, and the Widex AE using a linear frequency transposition [22]. It indicates that both provide improved audibility of high frequencies together with some signal distortion, but also result in considerably different percepts of the same original sounds.

The present study reports on tinnitus suppression over day and night for months to years, observed from hearing impaired subjects after several days or weeks of using daily the linear octave frequency transposition hearing aid.

## Materials and Methods

The data presented in this article were obtained over the last three years from two hearing aid dispensing centers. The selection of subjects for whom to try fitting with a linear octave frequency transposition (LOFT) hearing aid was based on the presence of a considerable hearing loss at high frequencies. About one third of these subjects had previous experience with another hearing aid but complained about it, for the other two thirds the LOFT aid was a first choice. Over the years the presence of invalidating tinnitus was also considered as a criterion for this choice. The patients were followed weekly during trials of different settings. Generally patients were given two settings of frequency transposition and were asked to alternate uses and appreciate their satisfaction over a period of at least two weeks. Although data logs in these hearing aids provided numbers of hours of use for the different settings which were collected into computer data bases, this large amount of data were not retrieved for analysis.

For each subject, basic medical informations were obtained at the first patient visit. In subsequent visits classical audiometric gains and speech intelligibility curves were measured and a brief description of patient satisfaction was also taken. For 38 subjects more precisely considered in this report, several tinnitus assessments were performed. The subjective level of tinnitus was obtained by using a visual analog scale (VAS) ranging from 0 to 10 in twenty steps. Localization of tinnitus was noted and approximations of its pitch and loudness were obtained by comparison with audiometer tones using reference frequencies of 2,3,4,6 and 8 kHz. For 31 subjects, estimates of tinnitus masking were also performed using the best tone-match frequency; for 12 of them masking was obtained at levels of 70 dB HL or more and for 13 of them hyperacusis prevented masking success.

The results presented here above were also examined exclusively for the 23 subjects with a history of noise exposure, all results were found similar.

All statistical tests and plots were performed using the SigmaStat SigmaPlot software version 12. In agreement with French legislation, data in this study were obtained by professional health workers on their own patients and were not transmitted to anyone else (monocentric study), in addition data were treated anonymously.

## Results

Among 74 subjects tested with LOFT amplification, long-term tinnitus suppression was obtained for 60 of them (81%). At present, long-term tinnitus suppression for day and night has been reported as permanent by all 60 subjects who use the LOFT aid daily; for our group of subjects this suppression spreads from some weeks to about two years and a half. As for etiologies of hearing impairment, subjects could be classified in six categories presenting different success rates, these success rates were statistically tested using the X-square and Fischer tests. For three subjects the etiology was chimiotherapy (antibiotic or anticancer drugs), among these all underwent tinnitus suppression (not statistically significant). Deafness occurred after surgery for 3 subjects, tinnitus suppression was successful for all 3 (not statistically significant). For 8 cases suffering from sudden deafness, tinnitus suppression was obtained in 2 cases only, this low rate was not found statistically significant likely due to the small number of observations but still suggests a real trend. For 8 subjects head trauma was identified as the cause of hearing impairment, for all of them tinnitus suppression was successful (p<0.001). Hereditary deafness was diagnosed for 15 subjects, long-term tinnitus suppression was successful for 12 of them a statistically significant proportion (p<0.003). Finally 37 subjects had a history of noise exposure and tinnitus suppression was obtained for 32 of them (p<0.001). As for subjects for whom tinnitus was not suppressed, we did not find any etiologic or audiometric special feature.

**Figure 1:**
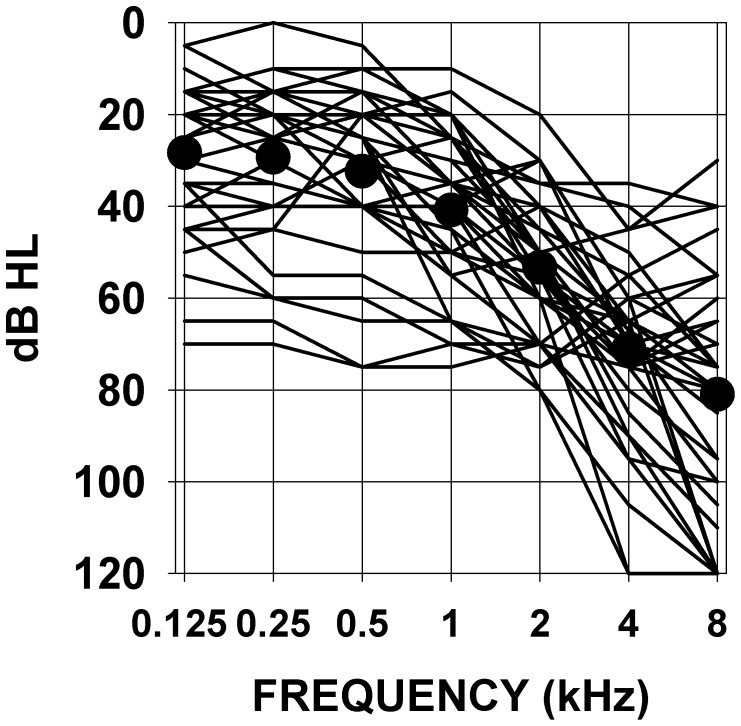
Audiograms of all the studied subjects. Abscissa : frequencies in kHz, ordinate : sound level in audiometric dB. Black circles : mean values.

**Figure 2:**
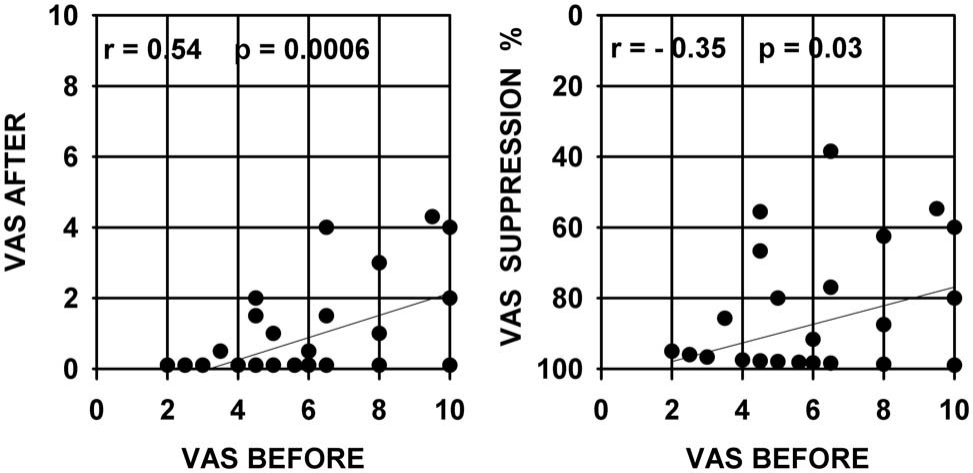
Subjective level of tinnitus before and after LOFT aid use. VAS : visual analog scale, values represent quantified subjective level of tinnitus. Abscissa : VAS score before LOFT aid use. Left graph : ordinate is VAS score after LOFT aid use. Right graph : same data but expressed as percent of suppression. Text in each graph indicates value of correlation coefficient (r) and statistical significance level (p).

**Figure 3:**
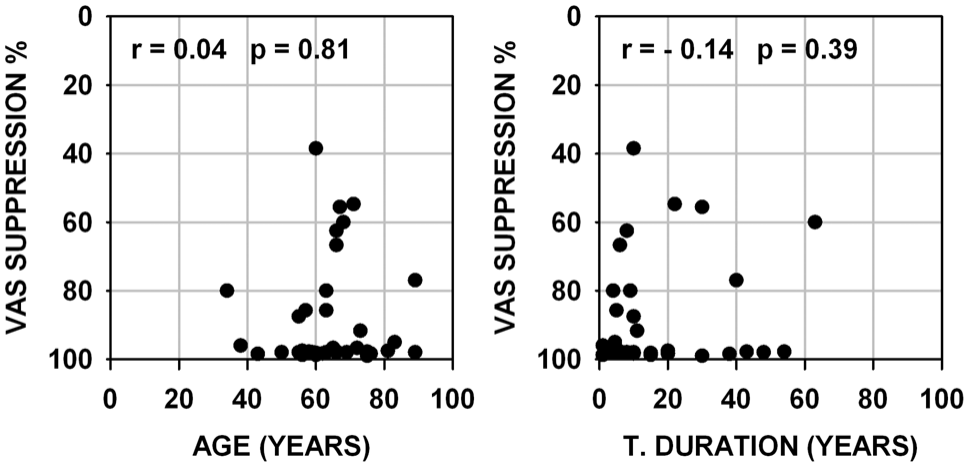
Percent of tinnitus suppression versus subject age or duration of tinnitus. Ratios of tinnitus suppression versus subjects age (left graph) and duration of tinnitus (right graph). Text in each graph indicates value of correlation coefficient (r) and statistical significance level (p).

**Figure 4:**
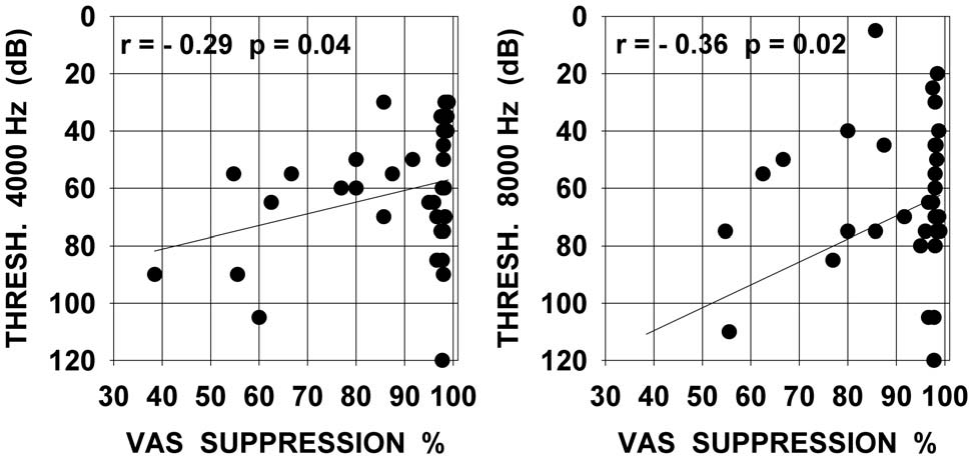
Percent of tinnitus suppression versus audiometric threshold at 4000 Hz and 8000 Hz. Abscissa : tinnitus suppression ratio (%). Ordinate : audiometric threshold in dB -left graph at 4000 Hz, -right graph at 8000 Hz. Text in each graph indicates value of correlation coefficient (r) and statistical significance level (p).

The more detailed data presented hereafter are restrained to 38 subjects for whom quantified measures of subjective tinnitus strength were obtained using a visual analog scale, and for whom several additional psychoacoustic and audiometric data could be collected. These 38 subjects presented somewhat similar audiograms with most often a rather steep hearing loss slope at high frequencies, all audiograms are presented in figure 1. The values of tinnitus strength as given by the subjects using a visual analog scale score before and after using a LOFT hearing aid are presented in figure 2 (left graph). The right graph shows the same data but in the form of percent of suppression obtained by the ratio : (VAS before minus VAS after )/VAS before which was then multiplied by 100. It can be seen from this figure that, over all individuals, tinnitus was subjectively evaluated as about half-suppressed to totally suppressed in the long-term. There is a significant correlation (Pearson product-moment correlation coefficient) between the subjective strength of tinnitus before LOFT use and the level of tinnitus suppression after LOFT use. The stronger the tinnitus was originally judged, the proportionally smaller was the percent of LOFT induced suppression. The correlation values are statistically significant but are not of high value and correspond approximately to 29–12% of the observed variability in the data. There was no correlation between the amount of tinnitus suppression and the age of the patient or the duration of tinnitus (figure 3); even for quasi life-long tinnitus complete suppression could be obtained. No correlation was observed between the level for tinnitus masking and/or the presence of hyperacusis and the amount of tinnitus suppression. Significant correlations were found between audiogram thresholds and subjective amount of tinnitus suppression. In figure 4 are presented graphs of threshold level versus amount of tinnitus suppression for frequencies of 4 and 8 kHz, frequencies to which tinnitus pitches were most often approximated. As seen in the figure, correlation values can be considered statistically significant but values are low; no correlation was found for 1 and 2 kHz thresholds but, unexpectedly, for 125, 250 and 500 Hz thresholds significant correlations were found but still with low values (respectively −0.44, −0.38 and −0.36). For each subject the high frequency slope of the audiogram was computed using a linear regression line approximation based on threshold values at 1,2,4 and 8 kHz, the slope value was expressed in dB per octave. No correlation was found between these slope values and the tinnitus VAS scores obtained. For all subjects an approximate pitch estimation of their tinnitus was obtained by comparison with audiometer tones, pitch estimates were matched by the subjects to frequencies of 2,3,4,6 or 8 kHz tones with a predominance for 4 kHz. No correlation was found between the amount of long-term tinnitus suppression and the frequency of the tinnitus pitch approximation. Tinnitus localization (right ear, left ear, bilateral or central) was also documented, suppression was found independent of tinnitus localization. Clearly tinnitus suppression was obtained in the LOFT aided ear but could also affect tinnitus in the opposite ear to various degrees but this point remains poorly documented in our data.

**Figure 5:**
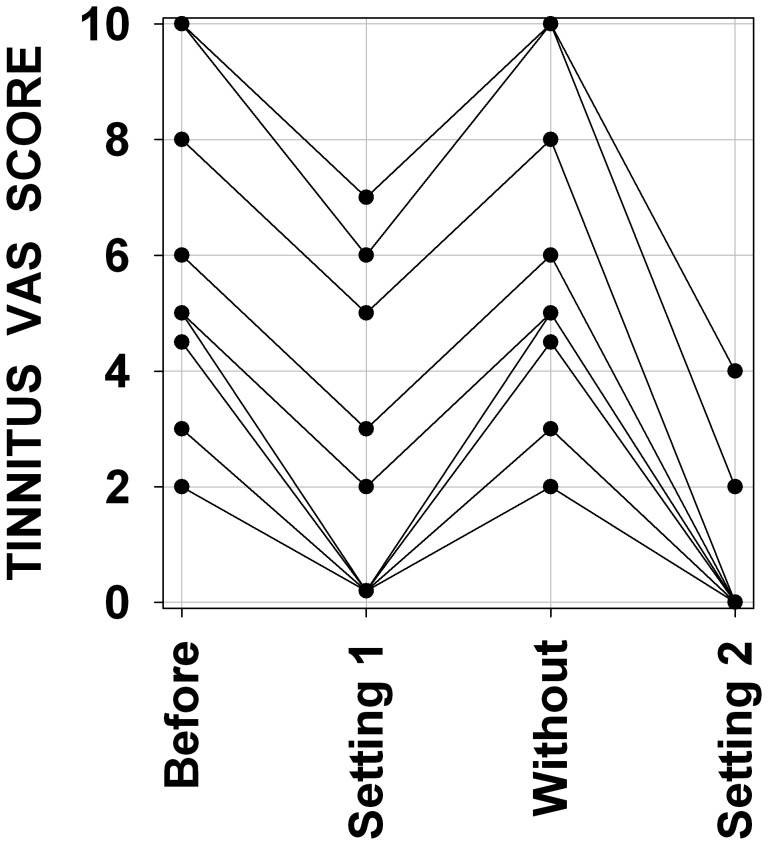
Variations of tinnitus subjective levels for 9 subjects in four conditions. Abscissa : subjective level of tinnitus. Ordinate : before LOFT aid use (Before), with a first frequency transposition setting (Setting 1), during a period without LOFT aid (Without) and with a second frequency transposition setting (Setting 2).

**Figure 6:**
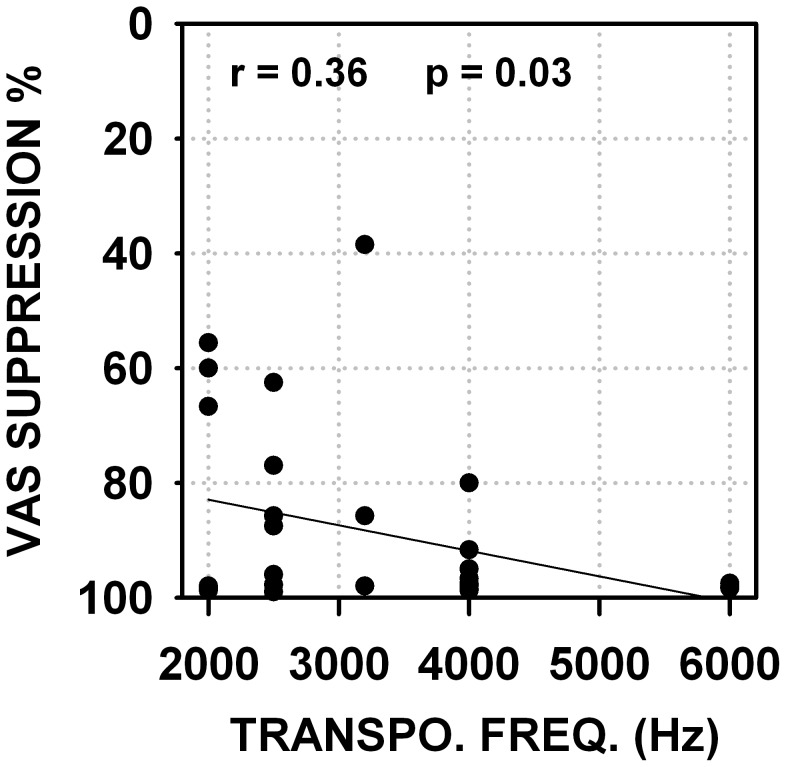
Percent of tinnitus suppression versus value of transposition frequency. Abscissa : value of transposition frequency, ordinate : tinnitus suppression ratio (%).Text in each graph indicates value of correlation coefficient (r) and statistical significance level (p).

The precise time course for the setting of long-term tinnitus suppression is generally poorly documented in our data because most subjects were not monitored on a fine time-base. However several subjects offered to provide a follow-up self-report. These subjects reported a long-term tinnitus suppression increasing over several days or weeks of LOFT aid use. For various reasons some subjects stopped using their aid for days to weeks, they all reported a reappraisal of their tinnitus within a day; when a LOFT aid was reused, the tinnitus suppression became effective again within a day of use. For several subjects two different amounts of frequency transposition trials were separated by a period of no aid use. Figure 5 presents VAS scores obtained in these different conditions for 9 subjects, it can be seen that in the period of no aid use subjective tinnitus strength returned to original values and that a different amount of frequency transposition could provide more tinnitus suppression. The amount of frequency transposition was empirically adjusted for each individual upon examination of the audiogram and mostly upon week trials and subject’s choice. The subjects reported their choice to be based upon best hearing as sounding most natural, and not upon tinnitus suppression. There is a weak correlation between the amount of frequency transposition and the strength of tinnitus suppression as can be seen in figure 6. We found no correlation between tinnitus pitch and most efficient amount of frequency transposition for tinnitus suppression.

## Discussion

It seems quite reasonable to think that tinnitus is often associated with auditory deprivation. Indeed for hearing-impaired persons complaining about tinnitus, an association with the audiometric loss seems to be most frequently observed [15], [16], [17]. Furthermore, normally-hearing subjects after some minutes in a silent cabin report tinnitus which resembles very much tinnitus described from complaints of hearing-impaired persons [3]. Tinnitus may then correspond to an abnormal neural activity associated with brain areas deprived of sound activation, hypothetically through alteration of lateral inhibition and/or synchrony and setting of hyper-reactivity presumably in link with hyperacusis often associated with tinnitus [23].

It also seems quite clear that reactivation of deprived sensori-neural areas can be associated with tinnitus suppression. Indeed there are many reports of tinnitus and temporary hearing loss from exposures to excessive sound levels showing that tinnitus disappears together with recovery of hearing loss [24], similar concomitances were made in many other pathological conditions such as Meniere’s disease, sudden deafness and drug ototoxicity. In cases of permanent hearing loss, there are suggestions that a reactivation of deprived areas can attenuate tinnitus [25].

In line with the two propositions above, we speculate that the tinnitus suppression reported in the present study could correspond to a perceptive re-attribution of transposed frequencies to auditory brain areas deprived by deafness. It might not be necessarily a re-excitation of deprived channels but it could act as a sort of gate mechanism. Because usual amplification and non linear frequency transposition did not provide similar tinnitus suppression for subjects of this study, both characteristics of linearity and of octave transposition may be of importance. Linearity allows conservation of harmonic relation – as spectral peaks remain at integer (although not necessarily successive) multiples-, which is well known as a strong factor for unifying components into a single auditory perception, preserving sound naturalness, a feature indicated by subjects in their choice of LOFT setting. In addition octave transposed components blend much better than components transposed at non octave ratios [26]. Remarkably in this line is the octave confusion often observed in tinnitus pitch matching [27] which may be an illustration of the perceptive strength of octave transposition. These two aspects may be critical for frequency transposed information to be perceptually fused with the un-transposed parts. Hypothetically once perceptually fused the transposed components might fit internal patterns used for recognition which would consequently re-attribute activity to deprived areas.
