# Correction: Long-Term Tinnitus Suppression with Linear Octave Frequency Transposition Hearing Aids

**DOI:** 10.1371/annotation/6d740ff6-82f0-4f33-a3e2-b0c9092c9baf

**Published:** 2013-01-15

**Authors:** Elisabeth Peltier, Cedric Peltier, Stephanie Tahar, Evelyne Alliot-Lugaz, Yves Cazals

The published Funding and Competing interests statements were incorrect. The correct statements are:

Funding: No current external funding was received for this study.

Competing interests: One or more of the authors are employed by Laboratoire Chelles surdité. This does not alter our adherence to all the PLOS ONE policies on sharing data and materials. 

